# Machine Learning-Based Models for Prediction of Critical Illness at Community, Paramedic, and Hospital Stages

**DOI:** 10.1155/2023/1221704

**Published:** 2023-06-26

**Authors:** Sijin Lee, Hyun Ji Park, Jumi Hwang, Sung Woo Lee, Kap Su Han, Won Young Kim, Jinwoo Jeong, Hyunggoo Kang, Armi Kim, Chulung Lee, Su Jin Kim

**Affiliations:** ^1^Department of Emergency Medicine, Korea University, College of Medicine, Seoul, Republic of Korea; ^2^Department of Industrial and Management Engineering, Korea University, Seoul, Republic of Korea; ^3^Department of Emergency Medicine, University of Ulsan, College of Medicine, Asan Medical Center, Seoul, Republic of Korea; ^4^Department of Emergency Medicine, Dong-A University, College of Medicine, Busan, Republic of Korea; ^5^Department of Emergency Medicine, Hanyang University, College of Medicine, Seoul, Republic of Korea; ^6^School of Industrial and Management Engineering, Korea University, Seoul, Republic of Korea

## Abstract

Overcrowding of emergency department (ED) has put a strain on national healthcare systems and adversely affected the clinical outcomes of critically ill patients. Early identification of critically ill patients prior to ED visits can help induce optimal patient flow and allocate medical resources effectively. This study aims to develop ML-based models for predicting critical illness in the community, paramedic, and hospital stages using Korean National Emergency Department Information System (NEDIS) data. Random forest and light gradient boosting machine (LightGBM) were applied to develop predictive models. The predictive model performance based on AUROC in community stage, paramedic stage, and hospital stage was estimated to be 0.870 (95% CI: 0.869–0.871), 0.897 (95% CI: 0.896–0.898), and 0.950 (95% CI: 0.949–0.950) in random forest and 0.877 (95% CI: 0.876–0.878), 0.899 (95% CI: 0.898–0.900), and 0.950 (95% CI: 0.950–0.951) in LightGBM, respectively. The ML models showed high performance in predicting critical illness using variables available at each stage, which can be helpful in guiding patients to appropriate hospitals according to their severity of illness. Furthermore, a simulation model can be developed for proper allocation of limited medical resources.

## 1. Introduction

Overcrowding of emergency department (ED) continues to be a problem faced by hospitals around the world [1–5]. This issue has strained national healthcare systems and healthcare workers in EDs. Furthermore, ED overcrowding adversely affects the clinical outcomes of critically ill patients [4, 5]. In circumstances where the ED is overcrowded, critically and noncritically ill patients compete for limited medical resources, and as a result, some critically ill patients may not receive proper medical services.

In South Korea, a three-level system of emergency medical institutes is available. They are classified into regional emergency medical centers (EMCs), local EMCs, and local emergency medical institutions. Regional EMCs and local EMCs provide 24-hour emergency care for critically ill patients, while local emergency medical institutions primarily care for noncritically ill patients. Reportedly, more than 75% of the annual ED visits in South Korea are made by vehicle rather than ambulance or walk-in, and regardless of their severity of illness, patients tend to choose a higher-level hospital such as EMCs or a nearby hospital [6, 7]. Such tendencies or uninformed choices can exacerbate the imbalance between the limited supply and overwhelming demand for medical resources.

Therefore, the early identification of critically ill patients prior to ED visits can help with the effective allocation of ED resources and prevent negative effects on patient outcomes [2–4]. If critical illness can be predicted at the community or paramedic stage, these data can help guide patients or paramedic ambulances to visit the appropriate level of emergency medical institutes according to the severity of their illness [8, 9]. Moreover, predicting and monitoring critical illness at the community or paramedic stage are crucial in establishing resource management policies, especially in situations where medical resource requirements are constantly increasing. Therefore, this study aimed to develop machine learning-based models to predict critical illness at the community, paramedic, and hospital stages using variables available at each stage.

## 2. Materials and Methods

### 2.1. Study Population and Data Sources

This study was conducted based on administrative data from Korean National Emergency Department Information System (NEDIS) (N20190320311). The NEDIS is a nationwide registry launched by the Ministry of Health and Welfare of Korea in 2003. A total of 414 EDs throughout Korea participated in the NEDIS, consisting of 36 regional EMCs, 118 local EMCs, and 260 local emergency medical institutions in 2019. Demographics and clinical information of patients visiting EDs is transmitted to NEDIS in a real-time basis. As patient information was anonymized and deidentified, the requirement for consent was waived. This study was approved by the Korea University Anam Hospital Institutional Review Board committee (No. 2019AN0263). From NEDIS data, adult patients (age ≥15 years) who visited EMCs from January 1, 2016 to December 31, 2017 were chosen as the study population since critically ill patients were focused on. Those who were dead on arrival (DOA), those who had Korea Triage and Acuity Scale (KTAS) at level 8 or 9 (others or unknown), and those who had missing values or invalid values (e.g., inconsistencies in vital signs based on the NEDIS guideline) were excluded.

### 2.2. Variables and Endpoint

The study utilized data from the public database in South Korea, and the variables included in the analysis were investigated as basic items within the NEDIS data. These variables included patient age, gender, triage measured by the KTAS, mode of arrival, ED visit date, ED visit time, chief complaint, symptom onset to ED arrival, systolic and diastolic blood pressure, pulse rate, respiratory rate, body temperature, mental status (AVPU), and number of diagnostic codes. The selection of variables was based on their availability at each stage and their relevance in predicting critical illness. Age, a consistent risk factor for critical illness, is associated with an increased need for ICU admission and higher mortality rates [10]. Vital signs, such as respiratory rate, systolic blood pressure, and heart rate, are predictive of critical illness and are used in clinical prediction models and triage tools such as the Emergency Severity Index (ESI) [11–14]. Mental status is a simple assessment tool with prognostic value in predicting critical illness [15]. Chief complaints, such as chest pain, dyspnea, mental change, and hematemesis, are associated with poor clinical outcomes [15, 16]. While the date and time of ED visits may not directly impact the severity of illness, they provide valuable contextual information that helps in understanding ED visit patterns and enhancing the performance of predictive models [17–19].

The emergency medical process was classified into three stages: (1) community, (2) paramedic, and (3) hospital. Variables were assigned according to their availability at each stage. The community stage consisted of variables such as age, gender, ED visit date, ED visit time, symptom onset time, chief complaint, and mental status. In the paramedic stage, vital signs were additionally included because paramedics could identify and measure them. The hospital stage encompassed all variables available in NEDIS data, including mode of arrival, KTAS level, and number of diagnostic codes.

In NEDIS data, chief complaints are recorded in Unified Medical Language System (UMLS) code using Korean medical terminology so that chief complaint can be mapped to the UMLS metathesaurus [20]. Due to a wide variety of UMLS codes, a panel of emergency medicine specialists was involved in categorizing chief complaints into 100 groups. Afterward, for each ED visit record, 100 separate binary variables with respect to 100 groups were newly defined as whether the patient had grouped chief complaint. As for diagnostic codes, we used International Classification of Diseases, Tenth Revision (ICD-10) codes from the NEDIS database.

The primary endpoint was “critical illness” that was defined as cases admitted to intensive care unit (ICU), transfer-out cases due to lack of ICU, death, or hopeless discharge at any point during hospitalization.

### 2.3. Development of Prediction Models for Critical Illness

Preprocessing of data were performed with R 3.4.1. This study applied random forest and light gradient boosting machine (LightGBM) among tree-based ensemble algorithms. Ensemble algorithms can improve the stability and accuracy of predictions by minimizing underfitting or overfitting in training data with high bias or variance. Ensemble algorithm-based learning methods include bagging (i.e., an acronym for bootstrap aggregating) and boosting. Bagging can reduce variance by training on a subset generated via random sampling of a dataset (i.e., bootstrap) and aggregating trained decision trees. Boosting also corresponds to an ensemble technique that can reduce both bias and variance by training hundreds or even thousands of weak trees. Random forest is a classic example of an ensemble model based on bagging. LightGBM is best known for its high performance based on boosting. Random forest trains the bootstrapped dataset with a bagging algorithm and finally predicts the data through voting.

The modelling of random forest and LightGBM was performed with *sklearn* and *lightGBM* packages in Python 7.8. The study population was split into a development dataset (70%) and a validation dataset (30%) at a 7 : 3 ratio using stratified random sampling. As performance measures, the area under the receiver-operating-characteristics curve (AUROC) and the area under the precision and recall curve (AUPRC) were computed. External validation of the prediction models was performed using the population that satisfied the same inclusion and exclusion criteria among ED visits registered in NEDIS from January 1, 2018 to December 31, 2018.

### 2.4. Variable Importance

Variable importance was calculated in random forest to gain insights into the contribution of each variable to the model for prediction of critical illness. The determination of variable importance is accomplished for each tree by randomly reordering the values of a single variable in out-of-bag samples and then putting the samples down each tree. After repeating this process for all variables (e.g., all bands) of one tree, the whole process is repeated for all trees in the forest. By measuring how much the model prediction changed, it is possible to estimate the importance of that variable.

## 3. Results

**Figure 1:**
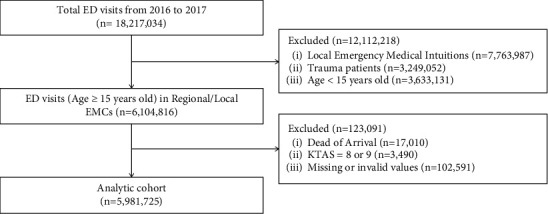
Flowchart of study population.

A total of 18,217,034 ED visits were collected in NEDIS during the study period. Among them, 6,104,816 adult ED visits in regional and local EMCs were identified. Those with DOA (*n* = 17,010), Korean Triage and Acuity Scale (KTAS) level 8 or 9 (*n* = 3,490) and those with missing or invalid values (*n* = 102,591) were excluded. Overall, 5,981,725 ED visits were included in study population (Figure 1). Critical illness during hospitalization occurred in approximately 5.77% of ED visits. Patients experiencing critical illness were older and more likely to be transported by ambulance, showed lower level of mental status, and presented with higher KTAS level (Table 1). Among patients with and without critical illness, median and interquartile range of vital signs were similar while total time from symptom onset to ED arrival was shorter in critically ill patients.

As for the prediction of critical illness, the performances of all models at three stages are shown in Table 2 and supplementary Figures S1–S3. In community stage, performances of LightGBM (AUROC: 0.877 (95% CI: 0.876–0.878) and AUPRC: 0.360 (95% CI: 0.358–0.363)) were slightly better than those of random forest (AUROC: 0.870 (95% CI: 0.869–0.871) and AUPRC: 0.353 (95% CI: 0.350–0.355)). In paramedic stage, LightGBM (AUROC: 0.899 (95% CI: 0.898–0.900) and AUPRC: 0.420 (95% CI: 0.417–0.424)) performed slightly better than random forest (AUROC: 0.897 (95% CI: 0.896–0.898) and AUPRC: 0.418 (95% CI: 0.415–0.421)). From the result of LightGBM, the predictability of critical illness in community stage, paramedic stage, and hospital stage were estimated to be 0.877, 0.899, and 0.950, respectively, based on AUROC.

**Figure 2:**
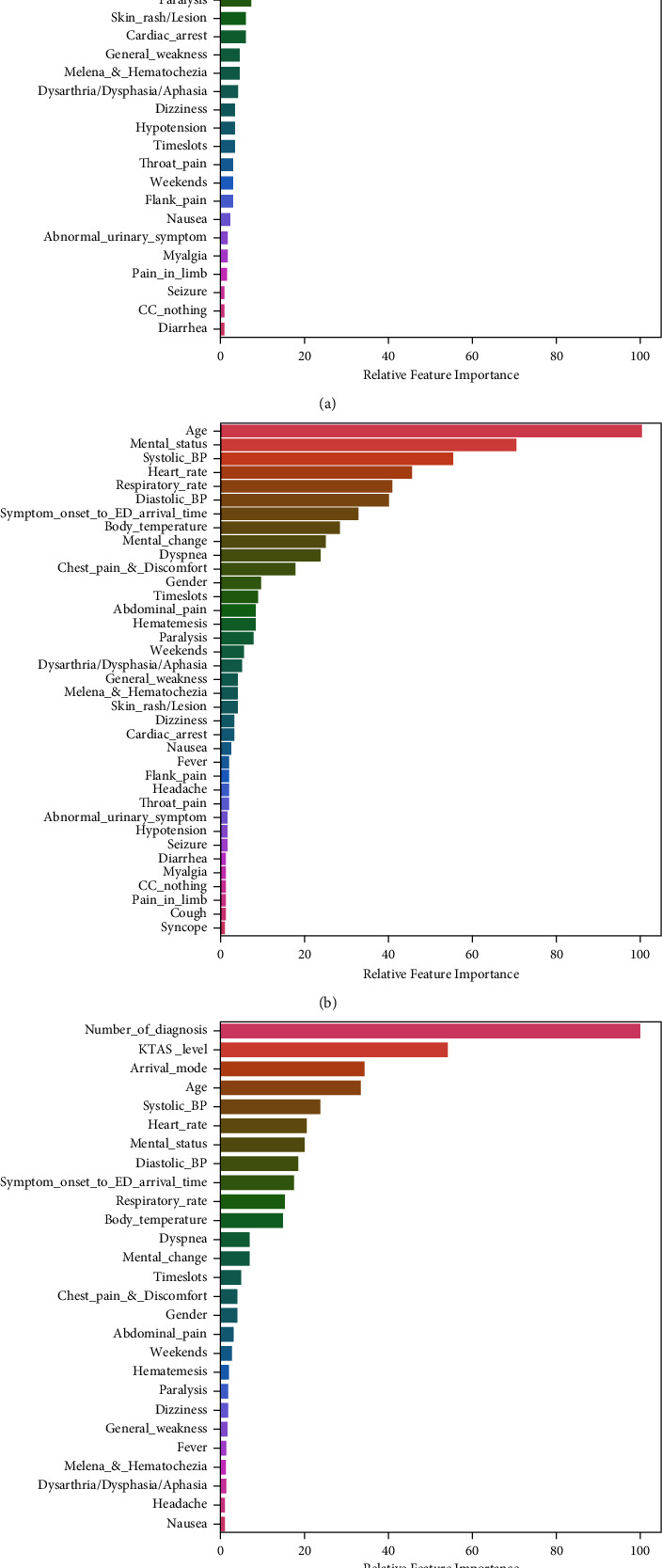
Variable importance for prediction of critically illness at community stage (a), paramedic stage (b), and hospital stage (c).

To gain insights into the relevance of each variable, the most important variables of random forest at each stage are summarized as shown in Figure 2. In community stage, variables such as age, mental status (AVPU), dyspnea, mental change, chest pain, hematemesis, symptom onset to ED arrival time, abdominal pain, gender, and paralysis were ranked in the top 10 important variables. In paramedic stage, vital signs were included in the upper ranks and showed higher importance than variables belonging to chief complaint or symptom onset time. In hospital stage, the number of diagnostic codes was the most important predictor, followed by KTAS level, arrival mode, age, and vital signs such as systolic BP and heart rate.

Table 3 shows recent machine learning studies to predict critical illness in the field of medical triage. All five studies utilized similar predictor variables, such as age, sex, chief complaints, vital signs, and comorbidities, similar to our study. The choice of models and their performance varied across these studies. Kang et al. achieved the best performance in terms of AUC, with a feedforward neural network (FFNN) model, registering an AUC of 0.867 (0.864–0.871). Other models, including random forest (RF), gradient boosting machines (GBMs), and deep neural networks (DNNs) were employed in these studies with varying degrees of performance. Machine learning-based prediction models consistently outperformed traditional models (ESI, LR, and NEWS) across all five studies.

**Figure 3:**
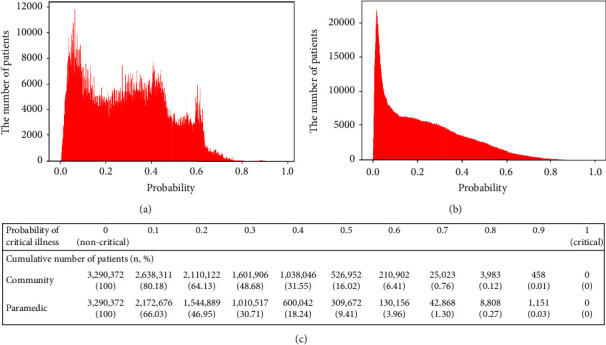
Probability distribution of critical illness in 2018. (a) Community stage, (b) paramedic stage, and (c) cumulative number of patients by probability of critical illness.

External validation of the LightGBM model for prediction of critical illness was conducted in the population that satisfied the same inclusion criteria and registered in NEDIS in 2018. The AUROC value of the predictive model was 0.841(95% CI: 0.840–0.842) in community stage and 0.874(95% CI: 0.873–0.874) in paramedic stage, showing similar performance in the external validation (Supplementary Table S1). The probability distribution of critical illness and cumulative number of patients by probability were also analyzed and are shown in Figure 3. The probability distribution of critical illness at community stage was skewed to the right and showed a mixed form of step and linear function, whereas at paramedic stage, the linear function was more prominent.

## 4. Discussion

In this study, ML models were developed to predict critical illness at the community, paramedic, and hospital stages using a national database. The models demonstrated high predictive power across all stages, even in the community stage where vital signs and triage scoring systems were not available. Our findings highlighted the top important variables, such as age, mental status, vital signs, chief complaints, and symptom onset, which are consistent with clinical rationality. For example, in the community stage, chief complaints such as dyspnea, mental change, chest pain, and hematemesis were ranked high in importance and these symptoms are recognized as severe by existing triage tools like ESI [12, 14]. In the paramedic stage, vital signs were included in the top 10 important variables, reflecting their clinical significance. In the hospital stage, additional factors such as the number of diagnostic codes, triage level, and arrival mode are commonly used in risk stratification and clinical decision-making [11–14]. Age had the highest variable importance in the community and paramedic stages and ranked 4th in the hospital stage, which indicates its significance in all stages. Geriatric patients frequently use critical care, and the increasing use of ICU services by geriatric patients in many countries [10] correlates with our findings.

The predictive ML models and variable importance analysis can assist healthcare providers in several ways. First, by continually updating and refining triage protocols based on these insights, healthcare providers can make more accurate and efficient assessments, leading to better patient outcomes. Second, the predictive models can help guide patient flow to appropriate facilities based on their assessed risk of critical illness, relieving overcrowding in ED, and optimizing resource allocation. Third, our study facilitates improved communication among healthcare providers across various stages, leading to more effective patient handoffs and care coordination.

The previous study, Christopher et al, predicted critical illness using out-of-hospital variables (e.g., age, sex, RR, SBP, HR, pulse oximetry, mental status, and nursing home location) and their model demonstrated good discriminative capacity (AUROC 0.77 (95% CI: 0.76–0.78)) [23]. However, the model showed significant errors in calibration such as overidentifying critical illness among those judged at high risk and underidentifying critical illness among those judged at low risk. These errors may occur due to the limitations of traditional analyses such as logistic regression because they assume that the effect of one predictor is not influenced by the value of another predictor. When this is not true and the value of one predictor alters effect of another, there is said to be an “interaction” between the 2 predictors, and those interactions can affect the study result or model performance [24, 25]. Our models were able to consider the interaction between these variables using machine learning techniques and showed a good performance in all stages and in external validation. Also, we categorized chief complaints into 100 groups under the supervision of emergency medicine specialists and applied those variables to ML models (e.g., stomach-ache, bellyache, and abdominal pain for Abdominal pain), with the expectation in improving the model performances.

Table 3 summarizes relevant machine learning studies to predict critical illness in prehospital settings. In comparison to our study, our ML-based prediction model demonstrated superior performance in the paramedic stage, with an AUC of 0.899 (0.898–0.900), surpassing the best-performing model in the other studies. Furthermore, our model's performance in the community stage, with an AUC of 0.877 (0.876–0.878), was either similar to or slightly higher than the AUCs of other models. This suggests that our model holds the potential to accurately predict critical illness in the community stage, where vital signs are not available, or to predict critical illness for ED visits made by nonambulance patients, who constitute 75% of annual ED visits. Our study, therefore, highlights the value of our models in effectively predicting critical illness in both paramedic and community stages compared to other studies.

Figure 3 displays the probability distribution of critical illness in community and paramedic stages, as well as the cumulative number of patients based on their probability of critical illness. By predicting and monitoring these probabilities and patient numbers in the prehospital stage, healthcare providers can effectively allocate patients to suitable hospitals according to illness severity. A simulation model can be developed and applied to help balance the demand and supply of medical resources, using a national monitoring system for health resources and service availability. For example, in a society with a probability distribution of critical illness as shown in Figure 3(b), if the demand and supply of medical resources are balanced when the ICU capacity of EMCs accounts for 4% of inpatients, the cumulative patient proportion at 0.6 can serve as a surrogate indicator, as the probability value of 0.6 corresponds to about 4%. If proportions of patients with probability values of 0.6 and 0.7 increase to 6% and 4%, respectively, hospitals can anticipate an increased demand for ICU care and prepare accordingly. With the increase in critically ill patients from 4% to 6%, hospitals can predict a 2% rise and proactively prepare necessary medical equipment, personnel, and ICU beds. If expanding medical resources are not feasible, EMCs might explore alternative strategies. One approach involves accommodating patients with a probability of critical illness of 0.7 or higher, which represents 4% of inpatients and is equivalent to current ICU capacity, while transferring patients with a probability of critical illness between 0.6 and 0.7 to EMCs in other regions or lower-level facilities. Another method includes raising the ICU hospitalization criteria for visiting patients so that patients with a probability of critical illness of 0.7 or higher are admitted to the ICU, while those with a probability of critical illness below 0.7 are admitted to acute care or general wards. By employing the simulation model, EMCs can predict the number of critically ill patients at the prehospital stage and respond with specific figures and goals when an increased demand for medical resources is expected.

In situations where the number of critically ill patients suddenly increases due to infectious diseases such as the COVID-19 pandemic, rapid estimations of anticipated medical resource demand are essential for enhancing hospital preparedness. The simulation model can predict such situations in the prehospital stage, enabling emergency medical systems and hospitals to swiftly adapt by implementing suitable strategies at each stage [26, 27]. At the community level, an effective approach involves reducing medical resource use for noncritically ill patients through temporary screening clinics or residential treatment centers [28, 29]. At the paramedic stage, maintaining constant communication between hospitals and paramedics concerning the probability of critical illness and available resources can induce optimal patient flow [30]. At the hospital level, surge capacity is crucial for hospital preparedness and early estimation of increased medical resource demand facilitates effective capacity expansion. Strategies may include increasing hospital beds, expanding ward spaces [31, 32], converting general wards to ICUs [33, 34], reducing bed occupancy rates by discharging selective admissions and noncritically ill patients in the ED [31, 35], and establishing designated hospitals and alternative medical facilities for efficient resource and personnel utilization [36, 37].

### 4.1. Limitations

This study has several limitations. First, since we used a national administrative data source, extensive clinic information such as free-textual nursing notes, laboratory and ambulatory exams, patient comorbidities, and relevant patient/family medical history could not be used for developing the predictive model. In the case of using high-dimensional or time-series electronic health records (EHRs) data, natural language processing (NLP) methods can be explored to extract meaningful information and further improve the predictive accuracy [38]. However, existing NLP methods are known to have limitations due to transcriptional inaccuracies (i.e., misinterpreting spoken words) and speech assignment errors (i.e., diarization) [39]. Chief complaint concepts can be handled with UMLS codes that contain a variety of information in a “source of knowledge” format. Thus, machine learning classification utilizing chief complaints based on UMLS codes allows predictive models to potentially have high performances [40].

Second, ICU admission was set as one of the definitions of critical illness, but hospitals may have different indications for ICU admission even if hospitals are of the same class. However, since many other studies for predicting critical illness also use ICU admission as a definition, it can be said that this is an academically acceptable range.

Lastly, our study population exhibited class imbalance, with critically ill patients constituting only 5.77%. Class imbalance can potentially skew the performance of predictive models, as machine learning algorithms tend to favour the majority class. To address this issue, we carefully selected machine learning algorithms such as random forest and LightGBM, which excel in handling imbalanced datasets [41, 42] and experimented with ensemble learning techniques, such as bagging and boosting, to enhance our model's overall performance [43, 44]. Additionally, it is crucial to note that the actual distribution of patients in ED is inherently imbalanced, and our dataset truly reflects this patient distribution. Although techniques such as oversampling or undersampling can be employed to mitigate the effects of imbalanced data [45], these methods have their limitations and may not always be feasible in real-world settings.

## 5. Conclusion

The ML models showed high performance in predicting critical illness using variables available in community and paramedic stages, which can be helpful in inducing patients to appropriate hospitals according to their severity of illness. A simulation model can be developed by monitoring probability of critical illness and the cumulative number of patients and can help health providers to respond more efficiently in proper allocation of limited medial resources.

## Figures and Tables

**Table 1:** Demographics and clinical characteristics between patients with critical illness and no critical illness.

Variables	Critical illness	No critical illness
Age (years), median (IQR)	70 (57–79)	51 (34–66)
*Gender*		
Female	141,957 (41.10)	3,048,163 (54.08)
Male	203,444 (58.90)	2,588,161 (45.92)
*Mode of arrival*		
Public/air-ambulance	129,754 (37.57)	951,941 (16.89)
Private ambulance	100,543 (29.11)	229,180 (4.07)
Police/public officer car/other transportation/walking	114,030 (33.01)	4,445,218 (78.87)
Others	1,061 (0.31)	9,741 (0.17)
Unknown	13 (0.00)	244 (0.00)
*ED visit date*		
On weekday	255,705 (74.03)	3,703,819 (65.71)
On weekend	89,696 (25.97)	1,932,505 (34.29)
*ED visit time*		
Night (0–8 hr)	52,871 (15.31)	1,225,776 (21.75)
Day (8−16 hr)	168,215 (48.70)	2,112,811 (37.49)
Evening (16−24 hr)	124,315 (35.99)	2,297,737 (40.77)
Symptom onset to ED arrival time (min), median (IQR)	347 (80−1,746)	494 (120−2,105)
*Mental status (AVPU)*		
Alert	258,331 (74.79)	5,508,812 (97.74)
Verbal response	38,168 (11.05)	80,213 (1.42)
Painful response	38,694 (11.20)	43,376 (0.77)
Unresponsive	10,208 (2.96)	3,923 (0.07)
*Vital signs*		
Body temperature (°C), median (IQR)	36.6 (26.2–37.0)	36.6 (36.4–37.1)
Heart rate (bpm per min), median (IQR)	90 (76–108)	82 (74–95)
Respiratory rate (per min), median (IQR)	20 (18–22)	20 (18–20)
Systolic blood pressure (mmHg), median (IQR)	128 (102–150)	130 (116–147)
Diastolic blood pressure (mmHg), median (IQR)	76 (60–90)	80 (70–90)
*KTAS level*		
Level 1 (resuscitation)	33,301 (9.64)	21,562 (0.38)
Level 2 (emergent)	140,934 (40.80)	385,822 (6.85)
Level 3 (urgent)	150,884 (43.68)	2,543,145 (45.12)
Level 4 (less urgent)	17,009 (4.92)	2,126,146 (37.72)
Level 5 (nonurgent)	3,273 (0.95)	559,649 (9.93)
Number of diagnostic codes, median (IQR)	4 (3–7)	1 (1–2)
Total	345,401 (5.77)	5,636,324 (94.23)

ED: emergency department; IQR: interquartile range; and KTAS: Korean Triage and Acuity Scale.

**Table 2:** Performance of machine learning-based prediction models at three stages.

Stage	Community	Paramedic	Hospital
*Random forest*			
AUROC	0.870 (0.869–0.871)	0.897 (0.896–0.898)	0.950 (0.949–0.950)
AUPRC	0.353 (0.350–0.355)	0.418 (0.415–0.421)	0.559 (0.555–0.562)
*Light GBM*			
AUROC	0.877 (0.876–0.878)	0.899 (0.898–0.900)	0.950 (0.95–0.951)
AUPRC	0.360 (0.358–0.363)	0.420 (0.417–0.424)	0.561 (0.558–0.564)

**Table 3:** Comparison of models for prehospital assessment to predict critical illness.

Authors	Data source	Predictors	Model	AUC (95% CI)
Lee et al. [21]	Single medical center	Age, sex, chief complaints, vital signs, and comorbidities	Neural network	0.801 (0.796–0.805)

Kang et al. [11]	NEDIS	Age, sex, chief complaints, vital signs, and symptom onset to arrival time	FFNN	0.867 (0.864–0.871)
			ESI	0.839 (0.831–0.846)
			NEWS	0.741 (0.734–0.748)

Shirakawa et al. [22]	Single medical center	Age, sex, chief complaints, and vital signs	LR	0.805 (0.782–0.827)
			RF	0.813 (0.786–0.834)
			GBM	0.818 (0.792–0.839)

Raita et al. [12]	NHAMCS	Age, sex, chief complaints, vital signs, comorbidities, and mode of arrivals	LR	0.84 (0.83–0.85)
			RF	0.85 (0.84–0.87)
			GBM	0.85 (0.83–0.86)
			DNN	0.86 (0.85–0.87)

Spangler et al. [13]	Uppsala ambulance service	Age, sex, chief complaints, vital signs, and operational characteristics of calls	GBM	0.79 (0.78–0.80)
			NEWS	0.76 (0.75–0.78)

AUC: area under the curve; CI: confidence interval; FFNN: feedforward neural network; ESI: emergency severity index; NEWS: national early warning score; LR: logistic regression; RF: random forest; GBM: gradient boosting machine; DNN: deep neural network; NEDIS: national emergency department information system; NHAMCS: national hospital and ambulatory medical care survey; and CHARS: comprehensive hospital abstract reporting system.

## Data Availability

The National Emergency Medical Center (NEMC) in Korea has administrative control and authority on the NEDIS (National Emergency Department Information System) data underlying this study. The NEMC review committee approves the research support proposed by researchers and provides deidentified NEDIS data to researchers for nonprofit academic research. Any researcher who proposes a study object and plans with a standardized proposal form and is approved by the NEMC review committee on research support can access the raw data. Detailed information on the approval process is now available on the NEMC website (https://dw.nemc.or.kr) or via contacting the NEMC review committee (skko@nmc.or.kr). The authors accessed the data used in this study in the same method that they expect other researchers to do so and did not receive special rights to access the data from the NEMC of Korea.

## Supplementary Materials

Supplementary Materials The external validation of the LightGBM model for prediction of critical illness in 2018 (Table S1). Receiver-operating-characteristics curve and recall-precision curve of random forest and LightGBM models for community, paramedic, and hospital stage (Figures S1–S3).
