# Supplementary material for: Machine Learning-Based Models for Prediction of Critical Illness at Community, Paramedic, and Hospital Stages
Source: Emerg Med Int. 2023 Jun 26;2023:1221704. doi: 10.1155/2023/1221704 (PMC10317605; doi:10.1155/2023/1221704)
Supplement: Supplementary Materials — The external validation of the LightGBM model for prediction of critical illness in 2018 (Table S1). Receiver-operating-characteristics curve and recall-precision curve of random forest and LightGBM models for community, paramedic, and hospital stage (Figures S1–S3). [file 1221704.f1.docx]

**Supplementary materials**

**Table S1. External validation of the LightGBM model for prediction of critically illness in 2018. A: Community stage B: Paramedic stage**

| **Year** | **Accuracy** | **Precision** | **AUROC** | **AUPRC** |
| --- | --- | --- | --- | --- |
| **A: Community stage** | | | | |
| 2016~2017 | 0.945 (0.945-0.946) | 0.61 (0.602-0.617) | 0.877 (0.876-0.878) | 0.36 (0.358-0.363) |
| 2018 | 0.850 (0.850-0.850) | 0.207 (0.206-0.208) | 0.841 (0.840-0.842) | 0.222 (0.220-0.224) |
| **B: Paramedic stage** | | | | |
| 2016~2017 | 0.947 (0.946-0.947) | 0.632 (0.627-0.638) | 0.899 (0.898-0.9) | 0.42 (0.417-0.424) |
| 2018 | 0.906 (0.905-0.906) | 0.297 (0.295-0.299) | 0.874 (0.873-0.874) | 0.326 (0.323-0.328) |


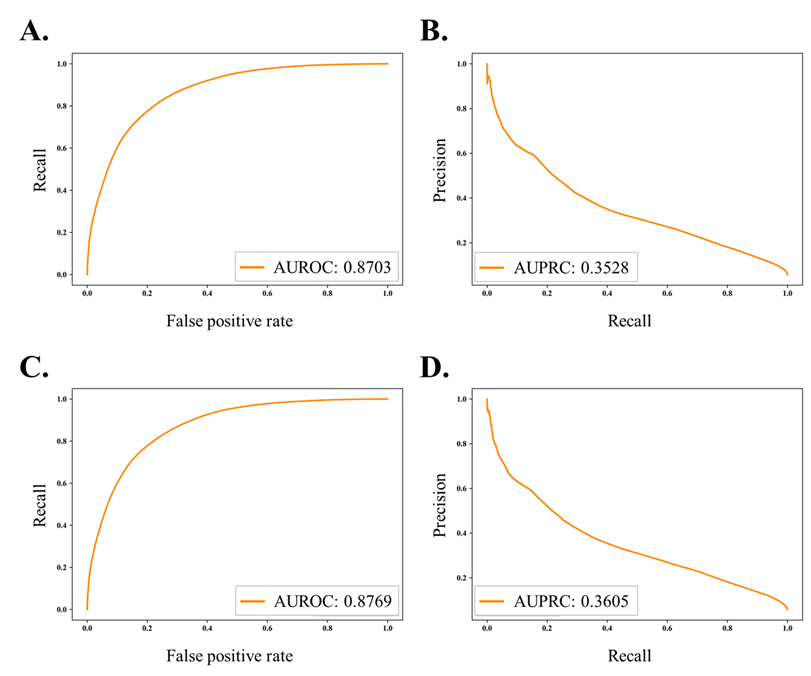


Fig S1. Receiver-operating-characteristics curve and recall-precision curve of Random Forest (A, B) and LightGBM (C, D) models for community stage


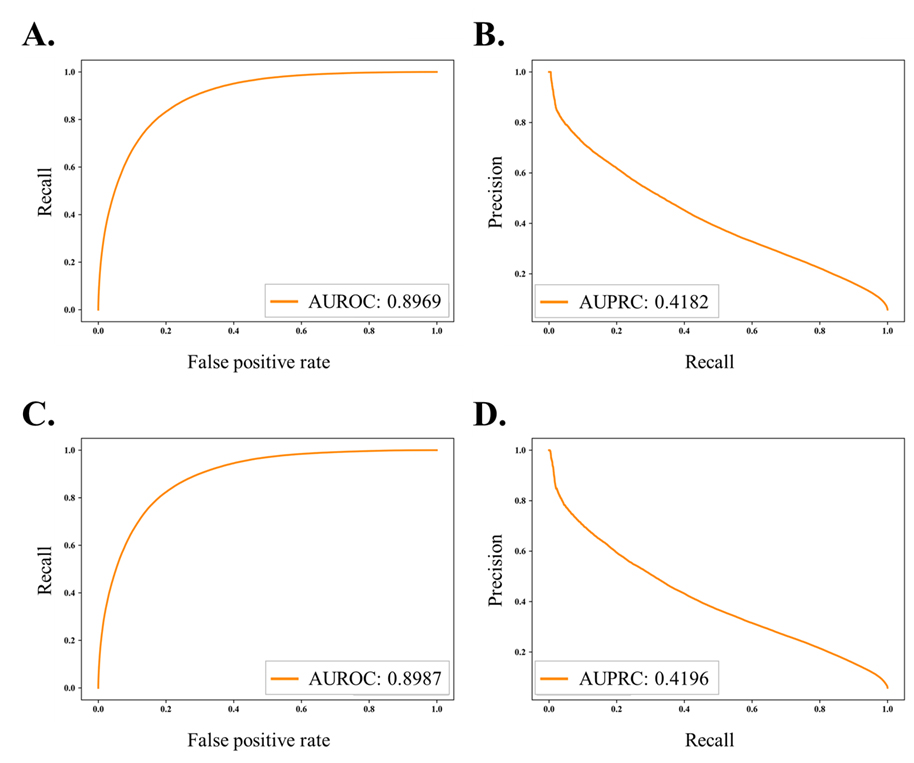


Fig S2. Receiver-operating-characteristics curve and recall-precision curve of Random Forest (A, B) and LightGBM (C, D) models for paramedic stage


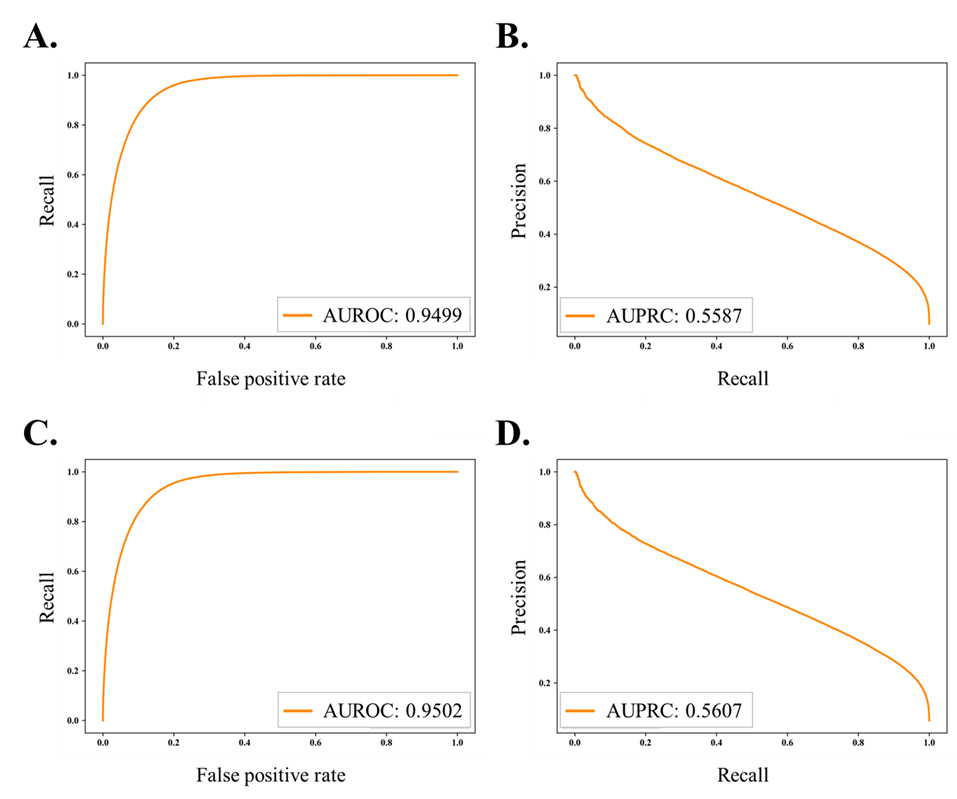


Fig S3. Receiver-operating-characteristics curve and recall-precision curve of Random Forest (A, B) and LightGBM (C, D) models for hospital stage
